# Evaluating the influence MRSA Co-infection on 28-day mortality among sepsis patients: insights from the MIMIC-IV database

**DOI:** 10.3389/fphar.2025.1534107

**Published:** 2025-03-11

**Authors:** Yi-Chang Zhao, Jia-Kai Li, Yu-kun Zhang, Zhi-Hua Sun, Rao Fu, Bi-Kui Zhang, Miao Yan

**Affiliations:** ^1^ Department of Pharmacy, The Second Xiangya Hospital, Central South University, Changsha, Hunan, China; ^2^ International Research Center for Precision Medicine, Transformative Technology and Software Services, Changsha, Hunan, China; ^3^ Xiangya School of Medicine, Central South University, School of Pharmacy, Changsha, Hunan, China; ^4^ School of Basic Medicine and Clinical Pharmacy, China Pharmaceutical University, Nanjing, Jiangsu, China

**Keywords:** MRSA, antimicrobial susceptibility, sepsis, 28-day mortality, MIMIC database

## Abstract

**Background:**

Sepsis remains a leading cause of mortality in intensive care units (ICUs), with methicillin-resistant *Staphylococcus aureus* (MRSA) infections presenting significant treatment challenges. The impact of MRSA co-infection on sepsis outcomes necessitates further exploration.

**Methods:**

We conducted a retrospective observational cohort study using the Medical Information Mart for Critical Care IV (MIMIC-IV-2.2) database. This cohort study included sepsis patients, scrutinizing baseline characteristics, MRSA co-infection, antimicrobial susceptibility, and their relations to mortality through Cox regression and Kaplan-Meier analyses.

**Results:**

Among 453 sepsis patients analyzed, significant baseline characteristic differences were observed between survivors (N = 324) and non-survivors (N = 129). Notably, non-survivors were older (70.52 ± 14.95 vs. 64.42 ± 16.05, P < 0.001), had higher lactate levels (2.82 ± 1.76 vs. 2.04 ± 1.56 mmol/L, P < 0.001), and higher SOFA scores (8.36 ± 4.18 vs. 6.26 ± 3.65, P < 0.001). Cox regression highlighted SOFA score (HR = 1.122, P = 0.003), body temperature (HR = 0.825, P = 0.048), and age (HR = 1.030, P = 0.004) as significant predictors of 28-day mortality. MRSA co-infection was found in 98.7% of cases without a significant effect on 28-day mortality (P = 0.9). However, sensitivity to cephalosporins, meropenem, and piperacillin/tazobactam was associated with reduced mortality. The area under the ROC curve for the combined model of age, SOFA, and body temperature was 0.73, indicating a moderate predictive value for 28-day mortality.

**Conclusion:**

While MRSA co-infection’s direct impact on 28-day sepsis mortality is minimal, antimicrobial sensitivity, especially to cephalosporins, meropenem, and piperacillin/tazobactam, plays a critical role in improving outcomes, underscoring the importance of antimicrobial stewardship and personalized treatment strategies in sepsis care.

## 1 Introduction

Sepsis, defined as a systemic inflammatory response syndrome characterized by extensive inflammation, organ dysfunction, and, in its most severe form, septic shock, remains a predominant cause of mortality within intensive care units (ICUs) globally (Catenacci et al., 2022; Laterre et al., 2019). Sepsis mortality is substantial at 20% worldwide, though this figure is improved from prior decades (Im et al., 2022). Recent scholarly investigations have revealed that the mortality rate among sepsis patients in ICUs can reach between 25% and 30%, thereby emphasizing the critical need for enhancements in therapeutic outcomes (Latronico and Herridge, 2015; Trof et al., 2010). In addition, after normalization to the population distribution in the 2010 Chinese census, sepsis-related mortality rate was 66.7% (Weng et al., 2018). Despite significant progress in medical technology and knowledge, the consistently high mortality rate associated with sepsis highlights the urgent need for ongoing research into effective management and therapeutic strategies. Besides, Methicillin-resistant *Staphylococcus aureus* (MRSA) sepsis is a severe condition associated with vascular leakage and poor prognosis (Lopez et al., 2021). MRSA is also responsible for around one-quarter of all deaths attributable to antibiotic resistance in high-income countries (AuthorAnonymous, 2023). Meanwhile, a study indicates that MRSA-sepsis triggers extensive proteome remodeling of the vascular cell surfaces, in a tissue-specific manner (Toledo et al., 2019). During MRSA infection, sepsis severity is linked to the bacterial a-toxin (a-hemolysin, AT) through unclear mechanisms (Luo et al., 2021). However, the intricacy of sepsis treatment is further compounded by the rising incidence of infections attributable to antibiotic-resistant bacterial strains (Plackett, 2022). Notably, the occurrence of MRSA in individuals suffering from sepsis is correlated with high rates of morbidity and mortality (Hassoun et al., 2017). Timely administration of antibiotics is one of the most important interventions in reducing mortality in sepsis (Im et al., 2022). However, antimicrobial resistance (AMR) is a global problem of increasing proportions that we cannot afford to look away from (Pariente, 2022). Additionally, studies also showed that AMR was a major and serious health problem globally in the world (Daneman et al., 2023; Murray et al., 2022). Given the substantial challenges posed by MRSA infections in the treatment of sepsis, this study endeavors to delve into the impact of MRSA co-infections on patient outcomes, with a specific focus on 28-day mortality rates. To achieve this objective, we leverage the Medical Information Mart for Critical Care IV (MIMIC-IV-2.2) database, which consists of electronic health record data from intensive care units in a tertiary care hospital in the United States (Lee et al., 2011; Saeed et al., 2011). This study concentrates on patients diagnosed with sepsis, encompassing those with confirmed MRSA infections. Its objective is to analyze baseline characteristics, outcomes, and the influence of MRSA co-infection and antimicrobial susceptibility on 28-day mortality. The findings aim to offer valuable insights into sepsis management amidst rising antibiotic resistance, informing future therapeutic approaches and antimicrobial stewardship initiatives.

## 2 Materials and methods

### 2.1 Database

The MIMIC-IV database, rich with demographics, vital signs, test results, and diagnoses coded using ICD-9 and ICD-10, was accessed by author Yichang Zhao, who obtained the required accreditation and extracted key variables for our study (ID: 11570656). This database ensures patient anonymity, eliminating the need for individual consent.

Our study utilized version 2.2 of the Medical Information Mart for Critical Care (MIMIC-IV), comprising 73,181 ICU records from 50,920 patients experiencing their initial ICU stay during hospitalization. We specifically analyzed first-time ICU admissions of patients 18 and older, excluding any ICU readmissions.

### 2.2 Study population and definitions

**FIGURE 1:**
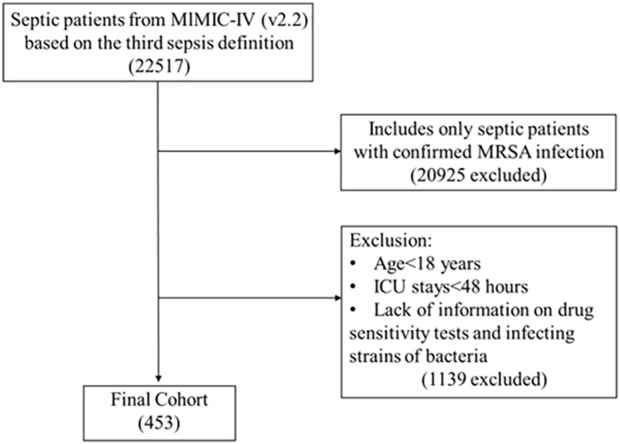
Study flow diagram in the present study.

Our study population included patients diagnosed with sepsis according to Sepsis 3.0 criteria and confirmed MRSA infection, as recorded in the MIMIC-IV database. Exclusion criteria were: (1) ICU stays shorter than 48 h; (2) multiple ICU admissions for sepsis, considering only the initial admission; (3) absence of relevant bacterial data; (4) diagnoses of diabetes mellitus or acute pancreatitis; and (5) treatment with lipid-lowering or antidiabetic medications, as shown in Figure 1.

### 2.3 Variable extraction

The primary exposure in our study was whether patients underwent MRSA drug sensitivity testing. Using SQL, we gathered baseline characteristics within the first 24 h of admission, including age, weight, ICU type, severity scores (SOFA, SAPS II, and Elixhauser Comorbidity Score), and vital signs such as MAP, heart rate, temperature, and respiratory rate. We also collected laboratory variables like WBC count, hemoglobin, platelet count, lactate, pH, PO2, and PCO2 during this period. Additional data recorded included the patient’s length of hospital stay, time to death, and results of drug sensitivity tests. For variables measured multiple times within the first 24 h, the value indicating the highest severity of illness was used.

### 2.4 Statistical analysis

Values are expressed as mean (standard deviation) or median [interquartile range (IQRs)] for continuous variables and as total and percentage for categorical variables. Comparisons between groups were made using the X^2^ test or the Fisher exact test and Student’s t-test for categorical variables or the Mann-Whitney U test for continuous variables (as appropriate). Multiple regression was used to characterize the association between MRSA infection and the primary outcome. Kaplan-Meier analyses and multivariate Cox regression were also used as sensitivity analyses to explore associations between MRSA susceptibility to different antimicrobials and MRSA co-infections on the 28-day mortality endpoint. All statistical analyses were performed using R version 4.1.2 (R Foundation) and IBM SPSS version 25.0. Statistical significance was defined as a two-sided P value <0.05.

## 3 Results

### 3.1 Baseline characteristics

We examined the baseline characteristics of 453 patients with sepsis, categorized into survivors (N = 324) and non-survivors (N = 129). The analysis revealed significant differences in several clinical and biochemical parameters between the two groups, which are summarized in Table 1 below. The average age was significantly higher in non-survivors (70.52 ± 14.95 years) compared to survivors (64.42 ± 16.05 years), with a P-value <0.001. Similarly, lactate levels were notably higher in non-survivors (2.82 ± 1.76 mmol/L) than in survivors (2.04 ± 1.56 mmol/L), also with a P-value <0.001, indicating that both age and lactate levels are critical factors associated with sepsis outcomes. Besides, significant differences were observed in platelet counts, with survivors having a higher count (229.52 ± 110.56 K/uL) compared to non-survivors (201.35 ± 106.79 K/uL), P = 0.013. Albumin levels were lower in non-survivors (2.83 ± 0.69 g/dL) compared to survivors (3.05 ± 0.67 g/dL), P = 0.012. Anion gap was higher in non-survivors (16.12 ± 4.41 mEq/L) than in survivors (14.85 ± 3.53 mEq/L), P = 0.004. In addition, markers of renal function such as BUN and creatinine, and liver enzymes (ALT, AST) differed significantly between survivors and non-survivors, indicating more severe organ dysfunction in the latter group.

**TABLE 1:** Comparison of baseline characteristics between survivor and non-survivor groups.

Categories	Survivor N = 324	Non-survivor N = 129	P-value
Age	64.42 ± 16.05	70.52 ± 14.95	<0.001*
Lactate (mmol/L)	2.04 ± 1.56	2.82 ± 1.76	<0.001*
Ph	7.36 ± 0.08	7.34 ± 0.10	0.072
Platelets (K/uL)	229.52 ± 110.56	201.35 ± 106.79	0.013*
WBC(K/uL)	13.33 ± 6.54	15.64 ± 10.05	0.09
Albumin (g/dL)	3.05 ± 0.67	2.83 ± 0.69	0.012*
Anion gap (mEq/L)	14.85 ± 3.53	16.12 ± 4.41	0.004*
Bun (mg/dL)	62.85 ± 42.31	78.12 ± 57.00	0.005*
Creatinine (mg/dL)	1.54 ± 1.39	1.75 ± 1.28	0.003*
Inr	1.54 ± 0.80	1.78 ± 0.93	0.001*
Pt	16.9 ± 8.57	19.16 ± 9.57	0.005*
Alt (U/L)	112.33 ± 404.27	204.72 ± 753.22	0.018*
Alp(U/L)	113.45 ± 105.02	124.73 ± 133.6	0.523
Ast (U/L)	208.69 ± 1041.49	328.15 ± 1025.74	0.004*
Total Bilirubin _	1.65 ± 3.84	2.22 ± 5.20	0.867
Sofa	6.26 ± 3.65	8.36 ± 4.18	<0.001*
Elixhauser	11.31 ± 10.69	15.94 ± 12.04	<0.001*

Meanwhile, non-survivors exhibited higher SOFA scores (8.36 ± 4.18) and Elixhauser comorbidity indices (15.94 ± 12.04), compared to survivors (6.26 ± 3.65 and 11.31 ± 10.69, respectively), both with P-values <0.001, suggesting that the severity of organ failure and the presence of comorbidities are predictive of mortality in sepsis.

### 3.2 Risk factor analysis for 28-day mortality in MRSA-infected septic patients

To identify significant predictors of mortality among sepsis patients, we conducted a COX regression analysis incorporating several clinical variables: SOFA score, age, direct bilirubin, leukocyte count, and BUN. The outcomes of COX regression is detailed in Table 2, reveal that three factors—SOFA score, body temperature, and age—significantly contributed to the risk of death among the patients studied. Specifically, each unit increase in the SOFA score was associated with a 12.2% increase in the hazard of death (Hazard Ratio [HR] = 1.122, P = 0.003), indicating the critical role of organ failure severity in predicting sepsis outcomes. Additionally, body temperature inversely affected mortality risk, with each degree Celsius increase associated with a 17.5% decrease in the hazard of death (HR = 0.825, P = 0.048), suggesting that lower body temperatures may be linked to higher mortality risks. Age also proved to be a significant factor; each additional year was associated with a 3% increase in the hazard of death (HR = 1.030, P = 0.004), highlighting the vulnerability of older sepsis patients.

**TABLE 2:** Binary logistic regression results for VRC hepatotoxicity.

Covariant	B	P Value	HR^a^	95% CI^b^
SOFA	0.144	0.003	1.122	1.039∼1.211
Temperature	−0.193	0.048	0.825	0.681∼0.999
Age	0.030	0.004	1.030	1.009∼1.052

^a^ OR, Hazard Ratio.

^b^ CI, confidence interval.

**FIGURE 2:**
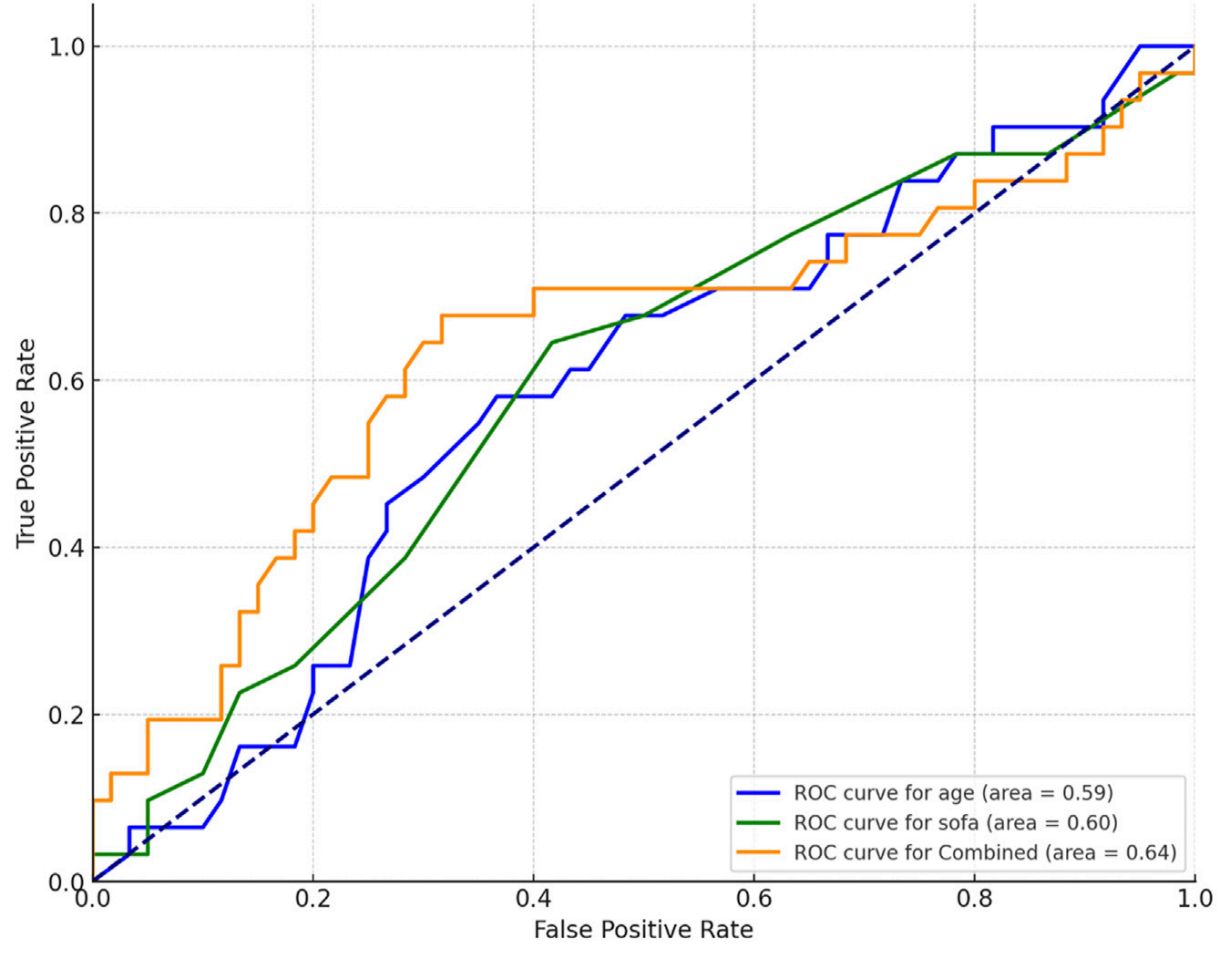
Receiver operating characteristic curve based on Age, SOFA and combined.

To assess the predictive capability of these metrics, Receiver Operating Characteristic (ROC) curves were plotted. The analysis highlighted the combined predictive impact of age and SOFA score, noting the minimal predictive value of body temperature alone (area under the ROC curve <0.5). As depicted in Figure 2, the ROC curve analysis emphasizes the substantial predictive power of these combined metrics for predicting mortality in sepsis patients, underscoring the clinical importance of age and severity of organ failure.

### 3.3 Impact of MRSA co-infection on patients’ 28-day mortality rate

**FIGURE 3:**
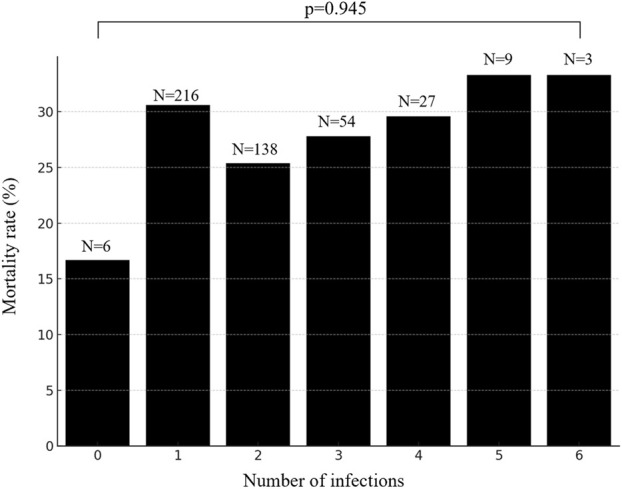
Number of co-infected pathogens in patients and mortality rate.

**FIGURE 4:**
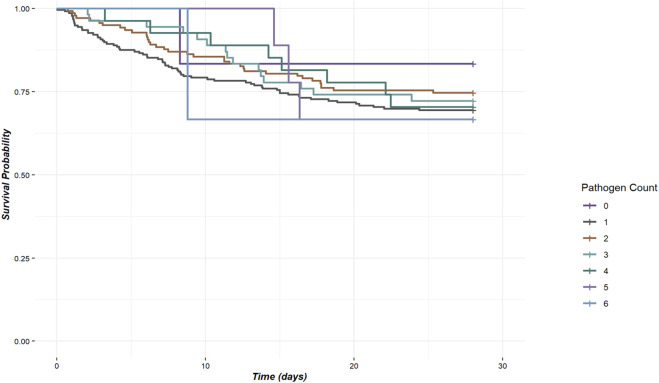
Kaplan-Meier survival curve curves for co-infections with different numbers of pathogens.

In our study of 453 patients, 98.7% (447/453) experienced co-infections, with the number of co-infecting pathogens ranging from zero to six, as shown in Figure 3. Detailed analysis revealed that patients with a single pathogen co-infection had a mortality rate of 16.7%. In contrast, those with dual or more pathogen co-infections exhibited increased mortality rates between 25.4% and 33.3%, with the rate peaking at 33.3% among patients co-infected with five to six pathogens (p-value = 0.945), as depicted in Figure 3. To further explore the impact of co-infections on survival, we conducted Kaplan-Meier survival analysis. The resulting curves, illustrated in Figure 3, compare survival probabilities across groups with different levels of pathogen co-infection. However, the number of co-infecting pathogens did not significantly affect the 28-day mortality rate (P = 0.9), as shown in Figure 4.

### 3.4 Effect of MRSA susceptibility or resistance to different antimicrobial drugs on 28-day mortality in patients

**FIGURE 5:**
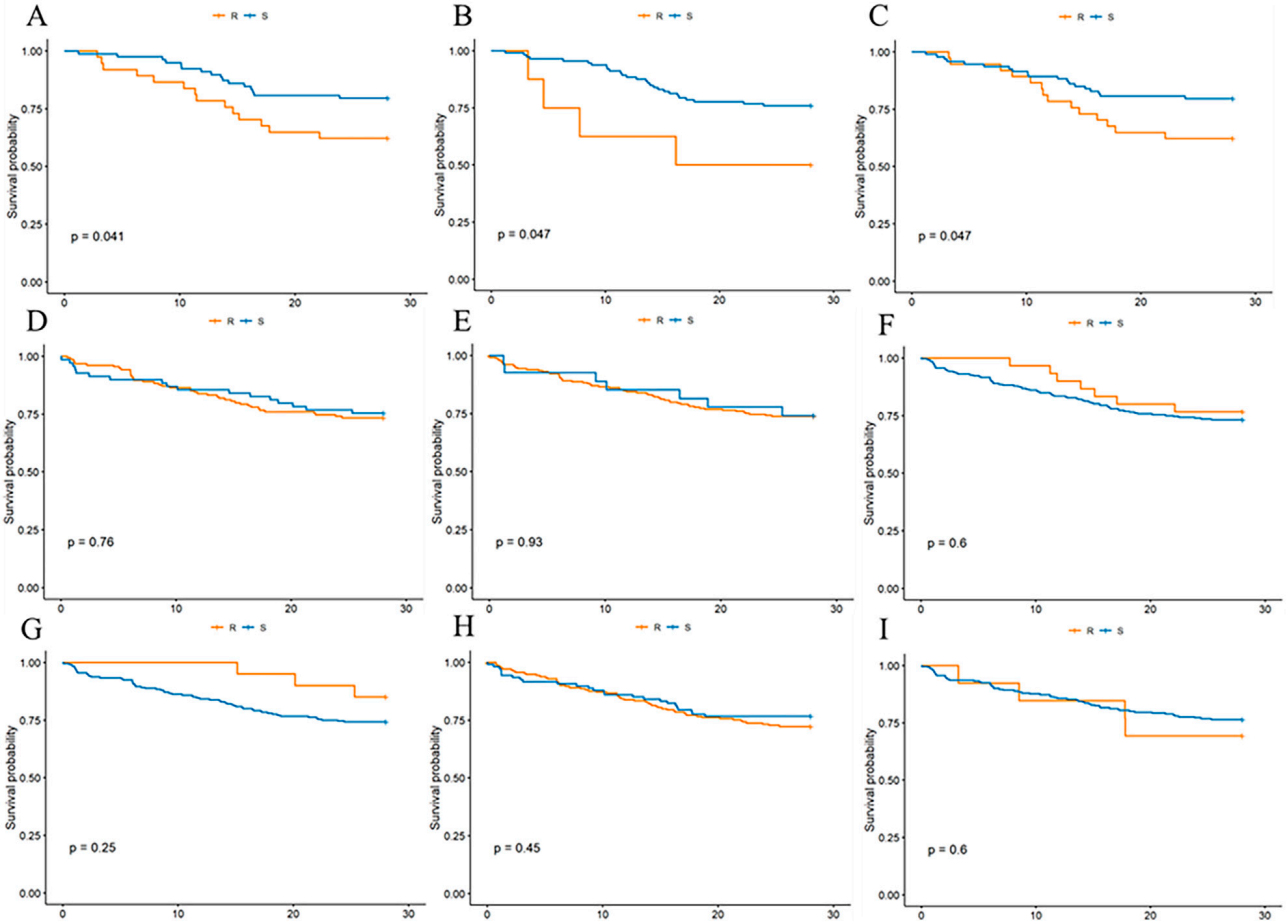
Effect of MRSA sensitivity or resistance to three classes of antimicrobial drug drug susceptibility tests on 28-day mortality in patients. **(A)** cephalosporins, **(B)** meropenemer, **(C)** piperacillin tazobactam/cefoperazone sulbactam, **(D)** clindamycin, **(E)** erythromycin, **(F)** TMP, **(G)** tetracyclines, **(H)** quinolones, **(I)** vancomycin.

To explore the impact of MRSA responsiveness to designated antimicrobials on 28-day survival rates in septic patients, we assessed MRSA sensitivity in a cohort of 453 patients. The survival curves showed that patients sensitive to cephalosporins (Figure 5A) had significantly higher survival rates than resistant patients, with a P-value of 0.047. Similarly, significant results were observed for meropenem (Figure 5B; P = 0.047) and the combination therapy of piperacillin-tazobactam/cefoperazone-sulbactam (Figure 5C; P = 0.041). These findings suggest that patients with MRSA strains sensitive to these antimicrobials had notably lower rates of 28-day mortality compared to those with resistant strains, as illustrated in Figure 5, highlighting the crucial role of antimicrobial susceptibility in treatment strategies for MRSA-associated sepsis. Additionally, sensitivity to other drugs like clindamycin, erythromycin, TMP, tetracyclines, quinolones, and vancomycin was analyzed, but these drugs showed no statistically significant difference in 28-day mortality rates among sensitive versus resistant patients.

### 3.5 Pathogen analysis of patient co-infections

**FIGURE 6:**
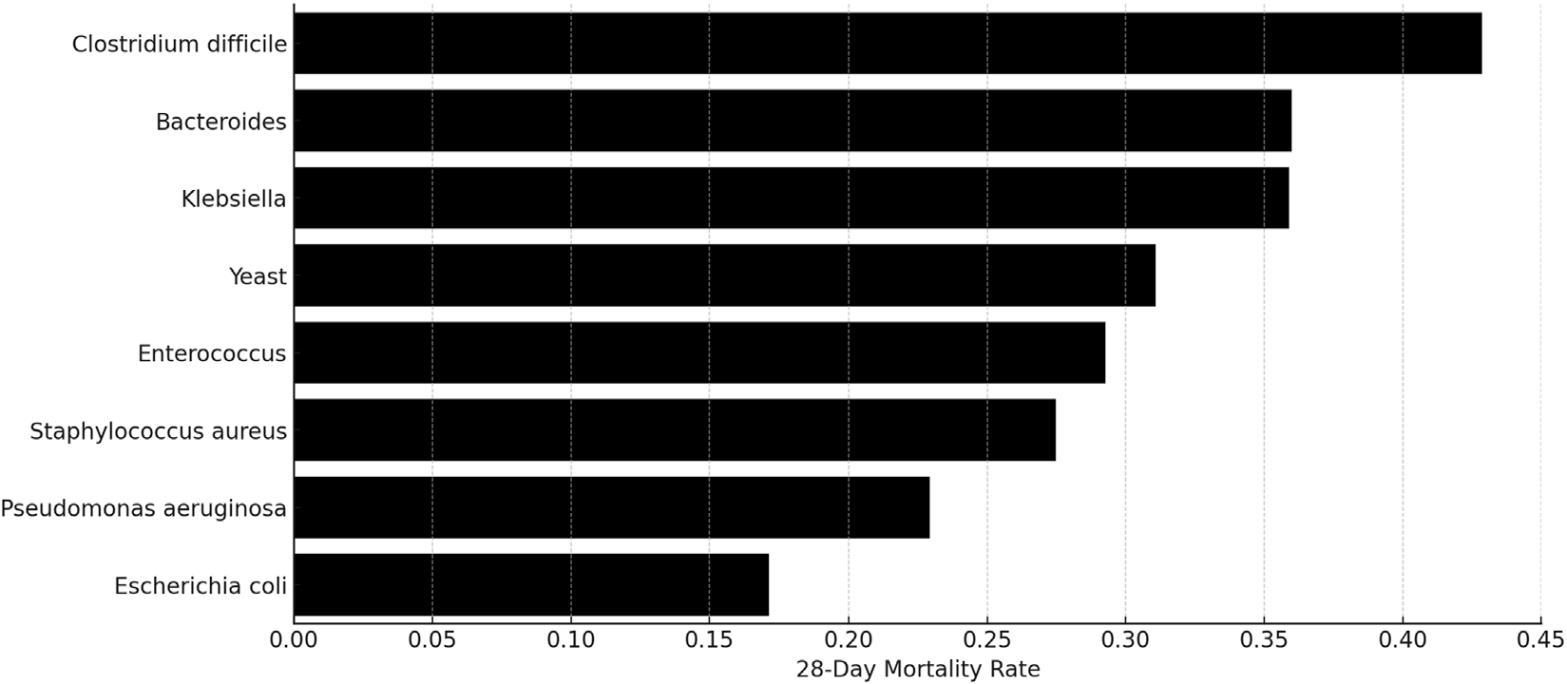
Mortality percentages among patients with MRSA and concurrent infections from other pathogen groups.

In our study, a high prevalence of co-infections was observed, with 447 out of 453 patients having multiple pathogenic infections alongside MRSA. We conducted a comprehensive analysis to assess the impact of these co-infecting organisms on the 28-day mortality rates. This analysis focused on pathogen groups with a minimum of 15 patients each (N ≥ 15). The results, shown in Figure 6, identified the eight pathogen groups with the most significant impact on mortality: *Clostridium difficile*, with a mortality rate of 43%; *Bacteroides* at 36%; *Klebsiella* spp. at 36%; Yeast at 31%; *Enterococcus* at 29%; *S. aureus* at 27%; *Pseudomonas aeruginosa* at 23%; and *Escherichia coli* at 17%.

**FIGURE 7:**
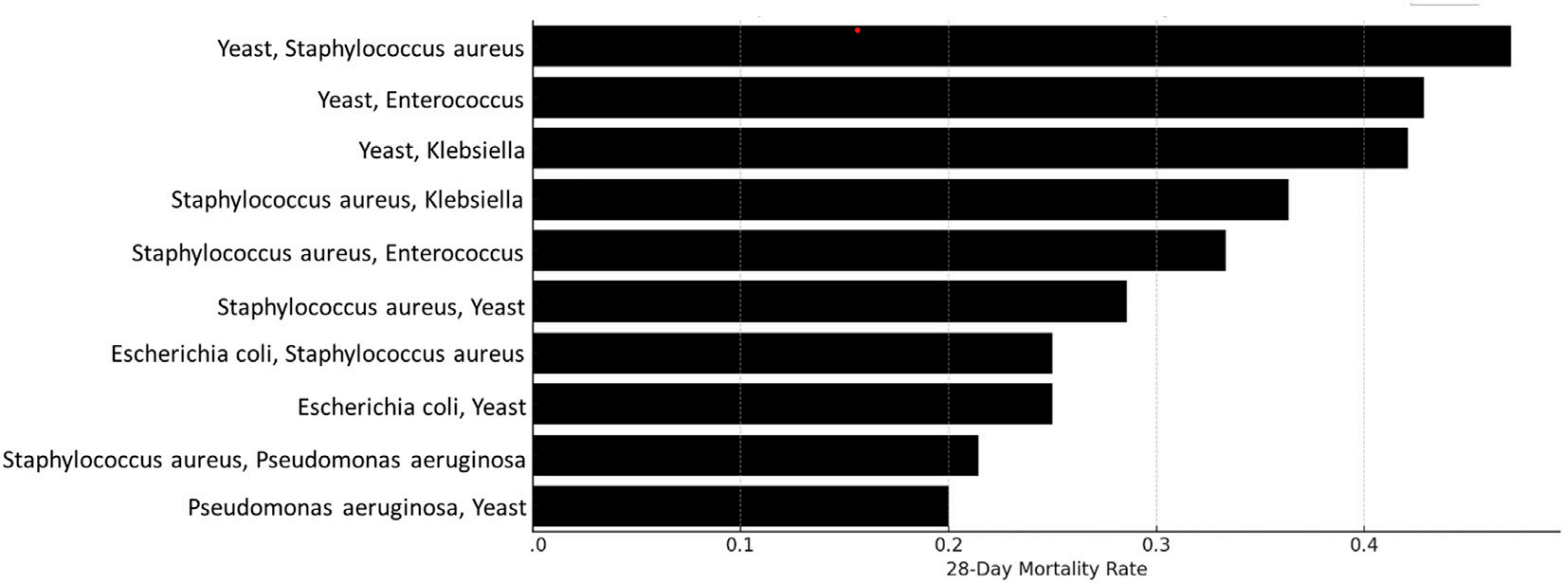
28-day mortality of patients co-infected with two pathogens.

Further analysis cataloged all pathogen presences among the 453 patients to identify specific combinations associated with increased mortality. This focused on subsets where patient counts exceeded ten. The mortality data for these co-infections are illustrated in Figure 7, which presents a clear depiction of the 28-day mortality rates across various groups in our study. The length of each bar indicates the mortality rate, with the highest bars representing the most severe outcomes. Remarkably, one group showed a mortality rate near 40%, significantly higher than others, which ranged from negligible to moderate.

## 4 Discussion

Drawing upon the comprehensive MIMIC-IV database, this study meticulously explored the impact of MRSA co-infection and antibiotic resistance on 28-day mortality in sepsis patients. By analyzing data from 453 ICU admissions, we elucidated significant roles of age, lactate levels, antibiotic susceptibility, and specific co-infecting pathogens in patient outcomes. Our investigation notably revealed that both age and lactate levels are significant prognostic markers for sepsis outcomes, aligning with other findings that emphasized the predictive value of lactate in sepsis mortality (Li et al., 2023; Baysan et al., 2020; Chaudhry et al., 2022; Liu et al., 2013; Schlapbach et al., 2017; Lee et al., 2021). Specifically, the non-surviving group had a mean age of 70.52 ± 14.95 years, significantly higher than the surviving group’s mean age of 64.42 ± 16.05 years, with a P-value <0.001. Elevated lactate levels, indicating metabolic distress, were notably higher in non-survivors (2.82 ± 1.76 mmol/L) compared to survivors (2.04 ± 1.56 mmol/L), with a P-value <0.001. These findings underscore the need for early recognition and aggressive management of sepsis in older patients and those presenting with high lactate levels.

The investigation into the role of antibiotic susceptibility testing in this study has elucidated its critical importance in guiding therapeutic decisions for sepsis management. Specifically, our analysis demonstrated that patients with MRSA infections who were susceptible to cephalosporins, meropenem, and the combinations of piperacillin-tazobactam/cefoperazone-sulbactam exhibited a markedly lower 28-day mortality rate. This finding signifies a substantial protective effect against mortality when antibiotic selection is informed by meticulous susceptibility testing. The concordance of our results with those of Daneman et al. (2023), who observed a similar protective effect in patients with *E. coli* bloodstream infections, reinforcing the generalizability of our findings across different types of bacterial infections. The consistency between these studies emphasizes the broader applicability of susceptibility testing-driven antibiotic therapy, not just for MRSA but also for other pathogens like *E. coli*, in reducing sepsis-related mortality. Furthermore, the importance of selecting the appropriate antibiotic therapy based on susceptibility testing is supported by several other studies (Hassoun et al., 2017; Lewis et al., 2018; Liu et al., 2011; Isler et al., 2022; Karampatakis et al., 2023a; Karampatakis et al., 2023b), which have corroborated the notion that tailored antibiotic strategies are vital in managing sepsis caused by MRSA or other bacteria. These corroborations suggest that a one-size-fits-all approach to antibiotic therapy in sepsis is suboptimal. Instead, an individualized approach, informed by rigorous susceptibility testing, is essential for improving patient outcomes in sepsis management.

Although the overall number of co-infecting pathogens did not significantly impact mortality rates, our deeper analysis into specific co-infecting organisms like Clostridioides difficile and *Klebsiella* spp. revealed an association with increased mortality. Thus, this observation highlights the crucial importance of not only focusing on the primary pathogen but also taking full account of the impact of co-infecting pathogens, especially those known to significantly affect prognosis. Similarly, Bekana K Tadese et al. found that infections with carbapenem-resistant Enterobacterales were associated with increased risk of death and polymicrobial infections with antimicrobial-resistance may add to the burden of clinical care and patients’ clinical prognosis (Tadese et al., 2022). Furthermore, Deng Pan et al. discovered that in instances of sepsis bloodstream infection concurrent with renal insufficiency, Gram-negative bacteria emerged as the primary pathogens. These infections proved to be more challenging to treat, necessitating a prolonged treatment course and the extended administration of antibacterial drugs (Pan et al., 2022).

In our study, we observed instances of MRSA infection patients in the MIMIC-IV database showing apparent sensitivity to cephalosporins, carbapenems, and β-lactam/β-lactamase inhibitor combinations, which is typically not expected given the known resistance patterns of MRSA. This discrepancy can likely be attributed to variations in antimicrobial susceptibility testing methods across different hospitals included in the database, as well as the potential for false-positive results due to differences between *in vitro* testing conditions and clinical realities. Additionally, the multi-center nature of the database and differences in data quality further complicate the interpretation of these results.

Despite the valuable insights provided, this study faces limitations due to its reliance on data from a single source, the MIMIC-IV database, which may limit the generalizability of the results. Additionally, the retrospective nature of the analysis might not account for all variables influencing sepsis outcomes. Future research should aim for multi-center and multi-regional studies to enhance the representativeness and applicability of the findings. Furthermore, the interaction between various pathogens and potential antibiotic cross-resistance necessitates further exploration to refine sepsis treatment protocols.

In summary, this study, utilizing the MIMIC-IV database, sheds light on the complex interplay between age, lactate levels, antibiotic susceptibility, and co-infecting pathogens on sepsis mortality. It calls for heightened attention to antibiotic resistance and advocates for precise diagnostic and therapeutic approaches to improve the care and outcomes of sepsis patients. Moving forward, continued research in this domain is essential to advance our understanding and develop more effective strategies against sepsis, ultimately aiming to reduce its global impact.

## Data Availability

Publicly available datasets were analyzed in this study. This data can be found here: https://mimic.mit.edu/.
